# Lyophilization Preserves the Intrinsic Cardioprotective Activity of Bioinspired Cell-Derived Nanovesicles

**DOI:** 10.3390/pharmaceutics13071052

**Published:** 2021-07-09

**Authors:** Yub Raj Neupane, Chenyuan Huang, Xiaoyuan Wang, Wei Heng Chng, Gopalakrishnan Venkatesan, Olga Zharkova, Matthias Gerhard Wacker, Bertrand Czarny, Gerrit Storm, Jiong-Wei Wang, Giorgia Pastorin

**Affiliations:** 1 Department of Pharmacy, National University of Singapore, Singapore 117559, Singapore; yub@u.nus.edu (Y.R.N.); chngweiheng@u.nus.edu (W.H.C.); gopal.venkatesan@smart.mit.edu (G.V.); matthias.g.wacker@nus.edu.sg (M.G.W.); 2 Department of Surgery, National University of Singapore, Singapore 119228, Singapore; huangchenyuan@u.nus.edu (C.H.); surwx@nus.edu.sg (X.W.); surzo@nus.edu.sg (O.Z.); g.storm@uu.nl (G.S.); 3 Cardiovascular Research Institute, National University Heart Centre, Singapore 117599, Singapore; 4 NUS Graduate School for Integrative Sciences and Engineering, National University of Singapore, Singapore 119077, Singapore; 5 Antimicrobial Resistance Interdisciplinary Research Group (AMR-IRG), Singapore-MIT Alliance for Research and Technology, Singapore 138602, Singapore; 6 School of Materials, Science and Engineering & Lee Kong Chian School of Medicine (LKC Medicine), Nanyang Technological University, Singapore 308232, Singapore; bczarny@ntu.edu.sg; 7 Department of Pharmaceutics, Utrecht Institute for Pharmaceutical Sciences (UIPS), Utrecht University, 3584 CS Utrecht, The Netherlands; 8 Department of Targeted Therapeutics, University of Twente, 7522 NB Enschede, The Netherlands; 9 Nanomedicine Translational Research Programme, Centre for NanoMedicine, Yong Loo Lin School of Medicine, National University of Singapore, Singapore 117609, Singapore; 10 Department of Physiology, National University of Singapore, Singapore 117593, Singapore; 11 NUSNNI-NanoCore, National University of Singapore, Singapore 117574, Singapore

**Keywords:** cell-derived nanovesicles, exosome mimetics, bionanotechnology, lyophilization, trehalose, cardioprotection

## Abstract

Recently, bioinspired cell-derived nanovesicles (CDNs) have gained much interest in the field of nanomedicine due to the preservation of biomolecular structure characteristics derived from their parent cells, which impart CDNs with unique properties in terms of binding and uptake by target cells and intrinsic biological activities. Although the production of CDNs can be easily and reproducibly achieved with any kind of cell culture, application of CDNs for therapeutic purposes has been greatly hampered by their physical and chemical instability during long-term storage in aqueous dispersion. In the present study, we conceived a lyophilization approach that would preserve critical characteristics regarding stability (vesicles’ size and protein content), structural integrity, and biological activity of CDNs for enabling long-term storage in freeze-dried form. Compared to the lyoprotectant sucrose, trehalose-lyoprotected CDNs showed significantly higher glass transition temperature and lower residual moisture content. As assessed by ATR-FTIR and far-UV circular dichroism, lyophilization in the presence of the lyoprotectant effectively maintained the secondary structure of cellular proteins. After reconstitution, lyoprotected CDNs were efficiently associated with HeLa cells, CT26 cells, and bone marrow-derived macrophages at a rate comparable to the freshly prepared CDNs. In vivo, both lyoprotected and freshly prepared CDNs, for the first time ever reported, targeted the injured heart, and exerted intrinsic cardioprotective effects within 24 h, attributable to the antioxidant capacity of CDNs in a myocardial ischemia/reperfusion injury animal model. Taken together, these results pave the way for further development of CDNs as cell-based therapeutics stabilized by lyophilization that enabled long-term storage while preserving their activity.

## 1. Introduction

In the field of nanomedicine, nanovesicles derived from cells of various origin have been explored for their potential as drug delivery systems [1,2], diagnostic probes [3], and cell-based therapeutics (i.e., with intrinsic pharmacological effects) [4,5,6,7]. The advantages of these naturally occurring vesicles (e.g., extracellular vesicles like exosomes) over their conventional counterparts (e.g., liposomes) include a lowered immunogenicity and innate targeting abilities, enabling them to be recognized and efficiently internalized by target cells [8,9,10].

Cell-derived nanovesicles (CDNs) represent a new class of cell-based vesicles obtained from subjecting cells to high shear forces using extrusion through sequential filter membranes of various sizes [11]. CDNs are lipid bilayer vesicles smaller than 200 nm in size, and possess—at least partly—the biological identity of the parent cells, i.e., they contain cell membrane proteins and lipids, metabolic enzymes, mRNA, and miRNA molecules originating from their parent cells [11,12]. One major advantage of the use of CDNs as compared to naturally secreted exosomes is a much higher production yield (in terms of protein content) and shorter processing time [11,13]. CDNs can be prepared from any type of cell using simple, scalable, and cost-effective production processes [11,13].

Despite the remarkable potential of CDNs as “exosome mimetics” in biomedical research, translation into clinical practice is still far away. From the pharmaceutical standpoint, the main drawbacks include limited physical stability (occurrence of denaturation, aggregation, and/or fusion) and chemical degradation (occurrence of hydrolysis, deamidation, and/or lipid oxidation) during long-term storage in the hydrated state (i.e., as an aqueous dispersion). In addition, there is still very limited knowledge of the in vivo fate of these nanovesicles upon administration as well as the factors which determine their biological behavior. Following the recommendations of the International Society for Extracellular Vesicles (ISEV) [14], cell-derived vesicles, including CDNs, must be stored at −80 °C to prevent the just-mentioned instability problems.

Nevertheless, Lorincz et al. reported that vesicle number and antibacterial activity of extracellular vesicles derived from human neutrophilic granulocytes significantly decreased during 28 days of storage at −80 °C [15]. Likewise, Maroto et al. reported substantial changes in the morphology of exosomes after 4 days of storage at −80 °C [16]. These studies clearly indicate that there is a compelling need to optimize the storage conditions for extracellular vesicles, including CDNs.

Lyophilization is an attractive approach to preserve the integrity of complex biological structures during long-term storage [17]. A phase transition of water from the frozen solid into the gaseous phase is achieved by evacuation of the drying chamber at reduced temperature. The primary drying or sublimation step is followed by secondary drying at elevated temperatures to support the desorption of water from the material surface. Thereby, residual moisture is reduced to 0.05 g water per g of dry powder sample [18]. Lyophilization has been extensively used to preserve the stability of nanosized conventional drug carriers including liposomes [19,20,21], lipid-based nanoparticles [22,23], and albumin-based nanoparticles [24]. This process has also been successfully employed to preserve the structure of biological materials such as DNA [18], RNA [17], and plasma [25].

However, what remains to be elucidated is whether lyophilization affects either the structural and/or functional properties of nanovesicles, as lyophilization is known to be accompanied by substantial mechanical stress during the freezing and drying processes [20], resulting in drastic changes in sample hydration level and pressure conditions [18]. Non-reducing disaccharides such as sucrose and trehalose have been extensively used to protect various biomolecules against the stresses generated during lyophilization via vitrification and/or water replacement hypotheses [26,27,28]. Hence, we hypothesized that lyophilization of CDNs in the presence of lyoprotectants such as non-reducing disaccharides could enhance the stability of CDNs by preserving their biomolecules, vesicular structure, and biological activity.

Therefore, the aim of this study was to assess the effect of the lyophilization process on the biomolecular structure properties of CDNs, as well as on their morphology and biological activity. Towards this goal, CDNs were produced from U937 monocytic cells as the CDNs from these immune cells were reported to have intrinsic targeting capability towards inflammation [29,30,31]. Moreover, U937 monocytic cell-derived exosomes were reported to carry several antioxidant enzymes (AOEs, namely SOD1, SOD2, catalase, GSTK1, and PRDX6) inherited from parent cells [12]. This suggests that CDNs from U937 monocytic cells should possess similar intrinsic activity (i.e., antioxidant capacity). By comprehensive in vitro and in vivo assays, we demonstrated that the biomolecular structures, intrinsic targeting capability, and biological activities of CDNs have been well preserved by lyophilization in the presence of an optimal concentration of trehalose.

## 2. Materials and Methods

### 2.1. Cells and Materials

U937 monocytic cells and CT26 mouse carcinoma cells were cultured in Roswell Park Memorial Institute (RPMI) 1640 medium supplemented with 10% fetal bovine serum (FBS). HeLa cells were cultured in Dulbecco’s modified Eagle’s medium (DMEM) supplemented with 10% FBS. Bone marrow-derived macrophages (BMDM) were obtained as previously described [32]. In brief, collected mouse bone marrow was filtered through a 0.45 µm filter and cultured in BMDM medium (80% DMEM and 20% FBS) supplemented with 30% L929 condition medium for 7 days to obtain BMDM. Spin cups pre-fitted with a 10-µm membrane filter were purchased from ThermoScientific, and 8 µm polycarbonate membrane was purchased from Merck Millipore, Singapore. Sephadex G-50 size exclusion gel, sucrose (molecular biology grade, ≥ 99.5% purity) and D-(+)-trehalose dihydrate (bioreagent grade, ≥ 99.0% purity) were purchased from Sigma–Aldrich, Singapore.

### 2.2. Production of CDNs from U937 Cells

CDNs were produced through a cell shearing approach using a spin cup as described by Goh et al. [11]. Briefly, 2 × 10^7^/mL U937 cells in 1× phosphate-buffered saline (PBS) were transferred to a spin cup pre-fitted with a 10-µm membrane filter and centrifuged twice at 14,000× *g* for 10 min at 4 °C. The flow-through was then transferred to another spin cup fitted with an 8-µm polycarbonate membrane and centrifuged twice at 14,000× *g* for 10 min at 4 °C. The CDNs dispersions were purified through Sephadex G-50 column equilibrated with PBS, whereby fractions third and fourth (containing 500 µL each) were collected and used for further experiments.

### 2.3. Characterization of CDNs

The hydrodynamic diameter and zeta potential of the CDNs were measured using the dynamic light scattering (DLS) technique and by determining the electrophoretic mobility, respectively (Malvern, Nano Series, Nano-ZS90). Concentrations of CDNs were determined using nanoparticle tracking analysis (Nanosight, NS300, Malvern instruments) at a wavelength of 405 nm. Protein concentration (expressed in µg/mL) was measured using bicinchoninic acid (BCA) assay (Pierce^TM^ BCA protein assay kit, ThermoScientific) in accordance with the supplier’s protocol, using bovine serum albumin (BSA) as a standard.

### 2.4. Lyophilization of CDNs

Sucrose or trehalose was added as lyoprotectant to the freshly prepared CDNs at varying sugar-to-protein ratios, with or without polysorbate 80, into 2-mL lyophilization vials. The concentrations of lyoprotectant and the ratios are summarized in Table 1. Samples were frozen at −80 °C for 8 h and were lyophilized using a bench-top manifold freeze dryer (FreeZone, Labconco) for 48 h at the vacuum pressure less than 0.1 mbar and condenser temperature below −50 °C. No secondary drying was performed. Lyophilized CDNs were reconstituted to their original volume with milli-Q water followed by gentle swirling for a few seconds before use.

### 2.5. Residual Moisture Content Determination

Residual moisture content was determined by the gravimetric method on a dry weight basis (g H_2_O per g of dry weight), by comparing with the initial sample weight (IW; g measured immediately after lyophilization) with dry weight (DW; g measured after heating in an oven at 80 °C for 12 h) [18]. Residual moisture content was determined using the following equation:

Residual moisture content (g H_2_O per g of dry weight) = (IW − DW)/Dry powder

### 2.6. Determination of Glass Transition Temperature

To determine the glass transition temperature (Tg), 5–10 mg of lyophilized CDNs was placed into a 40-µL aluminum pan, sealed hermetically, and placed into the sample holding chamber of the differential scanning calorimetry (DSC) instrument (Mettler Toledo DSC 1 STARe system) supplied with nitrogen flow at 50 mL/min. Simultaneously, an empty pan was used as a reference sample. The sample was firstly heated up from −20 to 120 °C to obtain a uniform sample and then cooled back to −20 °C. Tg was determined from the second heating scan from −20 to 150 °C, using a rate of 10 °C/min. A DSC thermogram between heat flow (w/g) vs. temperature (°C) was plotted and Tg was determined at the point of intersection by drawing the tangent line to the thermogram.

### 2.7. ATR-FTIR Spectroscopy

Fresh CDNs, as well as lyophilized samples (with and without lyoprotectant), were prepared and reconstituted with D_2_O to avoid the interference from -OH peak and phosphate peak from the buffer. Then the 10-µL sample was placed in a sample holder of attenuated total reflection Fourier transform (ATR-FTIR) spectrometer (Perkin Elmer, Spectrum 100) and scanned between 4000 to 650 cm^−1^ with 13 smoothing factors; spectra were averaged over 8 scans. A background scan of the clean sample holder was automatically subtracted, and the resulting spectra were analyzed using the spectrum software. The overall similarity between two spectra in terms of correlation coefficient (r) was calculated using the following equation [33]:

$$r=\frac{\Sigma X_{i}Y_{i}}{\sqrt{\Sigma X_{i}^{2}\Sigma Y_{i}^{2}}}$$

where *X_i_* and *Y_i_* represent the spectral absorbance values of the reference (fresh CDNs) and sample (lyophilized CDNs) spectra at frequency position *i*. For identical spectra, the r value is 1.0.

### 2.8. Far-UV CD Spectra Analysis

Secondary structure conformation and protein content associated with CDNs and lyophilized samples with and without lyoprotectant were determined using far-ultraviolet circular dichroism (Far-UV CD) spectrometry. Then 180 µL of the sample was probed using a quartz cell of 1 mm path-length in the CD spectrophotometer (Jasco J-1100 CD spectrometer) in the range of 190–260 nm. Detection was performed using detector PM-539 at a data interval of 0.1 nm, with a scanning speed of 100 nm/min at 20 °C. Spectra were collected from an average of 3 scans. A blank run of PBS was performed before sample analysis.

### 2.9. Cellular Association Study

All the samples used for cellular association and confocal microscopy analysis were labeled with Cyanine3 *N*-hydroxysuccinimide (Cy3-NHS) monoester as per the supplier’s recommendations. Lyophilized CDNs (S1, T1, and W) were labeled after redispersion in milli-q water, which maintained the same salt properties and pH of the PBS buffer before lyophilization. Labeled samples, which had a size of about 200 nm and a zeta potential of −7.37 mV, were dialyzed twice in dialysis cups (10 K MWCO; ThermoFisher Scientific) overnight in PBS.

For in vitro cellular uptake, 2.5 × 10^5^ HeLa or CT26 cells per well were seeded in 6-well plates, or 1 × 10^6^ BMDM per well were seeded in 12-well plates, incubated at 37 °C, 5% CO_2_. After 24 h, cells were treated with Cy3-labeled samples for 1 and 4 h. Then, cells were trypsinized and centrifuged at 450× *g* for 10 min. Cells were re-suspended in 500 µL PBS for analysis by BD LSR Fortessa flow cytometer at PE channel. Non-treated cells were used as control. In total, 10,000 events were analyzed, and data were processed using Flowjo software (Version 10.6, Ashland, Oregon, USA).

### 2.10. Confocal Microscopy

For confocal microscopy, 2.5 × 10^5^ HeLa cells were grown in a glass-bottom Petri dish in 2 mL of DMEM supplemented with 10% FBS for 24 h at 37 °C, 5% CO_2_. Then, cells were treated with Cy3-labeled samples for 6 h. Washed cells were stained with Hoechst 33,342 dye for 25 min to visualize nuclei and with CellMask deep red for 10 min to visualize the cell membrane. Imaging was performed using a laser scanning confocal microscope (FluoView, FV1000 Olympus) and images were processed using FluoView FV10ASW Version 4.2a software.

### 2.11. Measurement of Antioxidant Capacity

The total antioxidant capacity of CDNs, as well as lyophilized samples, was measured by total antioxidant capacity (TAC) colorimetric assay kit (BioVision, Inc., Milpitas, CA, USA) using Trolox as a reference standard, in accordance with the manufacturer’s protocol. Antioxidant enzymes (AOEs) superoxide dismutase (SOD), glutathione S-transferase (GST), and catalase present in the CDNs, as well as lyophilized samples, were measured using the assay kit (ab65354, ab65326, ab83464, respectively) as per the manufacturer’s protocol.

### 2.12. Long-Term Stability Study

Long-term stability of lyophilized CDNs was investigated in relation to the temperature of storage. CDNs normalized to a protein concentration of 530 µg/mL were lyophilized with sucrose (S1) or trehalose (T1), flushed with nitrogen gas, and kept for stability studies at 4 or 25 °C. Storage temperature was determined based on Tg values. At different time intervals (3 and 6 months), an aliquot was reconstituted and dispersed gently by swirling. The hydrodynamic diameter, protein concentrations, and total antioxidant capacity of CDNs were measured and the cellular uptake by HeLa and CT26 cells were analyzed by flow cytometry.

### 2.13. Evaluation of Cardioprotective Effect of CDNs

The cardioprotective effect of CDNs, as well as lyophilized CDNs in the presence of trehalose (T1), was examined in a mouse model of myocardial ischemia/reperfusion (I/R) injury as described previously [34,35]. In brief, male C57BL/6 mice (25–30 g) were subjected to left descending coronary artery ligation with 8–0 suture (Ethilon) for 30 min followed by removal of the ligature to allow myocardial reperfusion. CDNs or T1 (normalized to 40 µg of protein/100 μL) per mouse were injected intravenously 5 min before reperfusion, while 100 μL saline was injected to the control mice. After 24 h of reperfusion, mice were terminated and the heart tissue was used for 2,3,5-triphenyltetrazolium chloride (TTC) staining to determine the infarct area (IA) as a percentage of area at risk (AAR). All surgical procedures were approved by the National University Singapore Institutional Animal Care and Use Committee (IACUC) (approved protocol number R18-1452) and conformed to the guidelines on the care and use of animals for scientific purposes (NACLAR, Singapore, 2004) and the Guide for the Care and Use of Laboratory Animals published by the US National Institutes of Health (NIIH Publication, 8th Edition, 2011).

### 2.14. Statistical Analysis

Statistical analyses were performed using GraphPad PRISM 5 (version 5.01). Comparisons between multiple samples were performed by one-way ANOVA using Bonferroni post hoc test, where *p* < 0.05 was considered significant.

## 3. Results and Discussion

### 3.1. Preparation and Characterization of CDNs

**Scheme 1:**
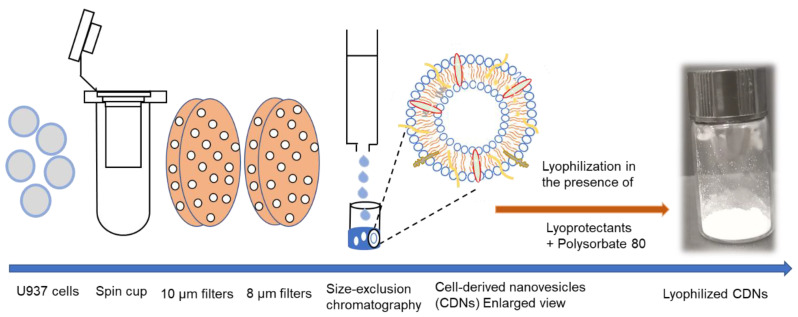
Schematic depiction of CDN production. U937 cells were passed twice through a spin cup fitted with 10- and 8-µm polycarbonate membranes by microcentrifugation (14,000× *g*) and CDNs were purified using size-exclusion chromatography. CDNs were subsequently lyophilized in the presence of various lyoprotectants to result in a uniform cake/powder.

The method for preparing CDNs from U937 monocytic cells is presented in Scheme 1. The vesicles exhibited a mean size of approximately 124.8 ± 8.4 nm (Table 1), displaying a spherical structure as indicated by the transmission electron micrographs (Supplementary Figure S1A). CDNs had a zeta potential of around −7 mV, the protein concentration of ~538 µg/mL, and concentration of ~3 × 10^10^ vesicles per mL. In our previous study, we demonstrated that CDNs mimicked exosomes derived from U937 cells in size, shape, and lipid composition, and expressed key marker exosomal proteins such as CD9, Alix, and TSG 101 [11]. This similarity with exosomes would suggest that functional aspects, such as specific binding and cellular uptake by target cells, are also preserved. This paper aimed to assess whether lyophilization is possible without impairing these structural and functional features.

### 3.2. Impact of Lyoprotectant on the Lyophilization of CDNs

To maintain vesicle structure and integrity of membrane proteins of CDNs as properties that enable recognition and subsequent internalization of CDNs by target cells, trehalose or sucrose were added as lyoprotectants during the lyophilization process. The crucial role of lyoprotectants for the stabilization of CDNs during the lyophilization process was confirmed by an increase in the hydrodynamic diameter in absence of either of the two disaccharides (from 124.8 ± 8.4 to 394.9 ± 30.9 nm; *p* < 0.001). During the freeze-drying process, sugars provide a matrix that inhibits molecular motions and stabilizes molecular structures by replacing the solvent molecules as binding partners.

Non-reducing disaccharides have been reported to be suitable excipients [18,20,28]. The matrix effect, or vitrification, is based on the ability of the lyoprotectant (mainly sugar) to form an amorphous, highly viscous glassy matrix in which particulate structures are embedded (Supplementary Figure S2). It results in a significant reduction in degradation reaction kinetics [36]. On the other hand, the replacement of bound water molecules was expected to lead to a thermodynamic stabilization of for example the CDNs’ membrane proteins due to the interaction of hydroxyl groups of the sugars with proteins and phospholipid headgroups. By replacing hydrogen bonds between water molecules and proteins, as well as between water molecules and phospholipid headgroups [20,28], the proteins’ native conformation can potentially be preserved during lyophilization (Supplementary Figure S3).

Without the lyoprotectant, proteins and other CDN components likely aggregate during lyophilization, as suggested by the increased size in our experiment. In addition, the total protein amount was significantly reduced (by 14.6%; *p* < 0.001) upon lyophilization when compared to the freshly prepared CDNs (Table 1). This indicates that the shear forces applied during lyophilization can induce a partial degradation of the proteins present in the CDNs. Even though degraded proteins might still provide a signal at the BCA protein quantification assay (thus suggesting an underestimation of the actual protein degradation), the heated samples were also associated with a change in proteins’ secondary structure. Noteworthy, a loss of protein activity is known to occur and has been extensively reported when samples were lyophilized in absence of a lyoprotectant [37,38,39,40].

Upon reconstitution, non-lyoprotected CDNs were not completely re-dispersible in milli-Q water and exhibited visible aggregation. These results suggest that the lyophilization process without lyoprotectant leads to considerable damage to the CDNs.

Upon addition of the non-reducing disaccharides sucrose or trehalose, aqueous dispersions of CDNs were lyophilized over 48 h at a lyoprotectant-to-CDNs’ protein ratio of 100:1 (*w*/*w*). Reconstituted CDNs, lyophilized either with sucrose or trehalose, however, showed a significant (*p* < 0.001) increase in the hydrodynamic diameter and a significant reduction in protein concentration as compared to CDNs before lyophilization (*p* < 0.001) (Table 1). These changes indicate that the lyoprotectants alone could not prevent vesicles from aggregation or fusion through the freezing and drying steps of the lyophilization.

It has been reported that non-ionic surfactants (e.g., polysorbate 80 or polysorbate 20) in the presence of lyoprotectant can preserve the biological activity of protein-based therapeutics [41] and can also provide steric stabilization to nanoparticles [42,43]. Therefore, in this study, the non-reducing disaccharides sucrose or trehalose were added in combination with polysorbate 80. Interestingly, even at a very low concentration (0.01% *w*/*v*) of polysorbate 80 added, CDNs could maintain the hydrodynamic diameter below 200 nm (Table 1). This may be due to the protecting effects of non-ionic surfactants such as polysorbate 80 against interfacial stresses that induced aggregation of CDNs during lyophilization. Moreover, binding of the hydrophobic segment of polysorbate 80 to the hydrophobic region of CDNs’ proteins may prevent protein self-association during lyophilization and storage [44,45]. Another potential mechanism is that polysorbate 80 preferentially coats exposed hydrophobic segments and thereby prevents the proteins from unfolding at the surface, thus maintaining their functional properties [44]. To discern the protective contribution of polysorbate 80 from one of the lyoprotectant disaccharides, we further assessed the effect of polysorbate 80 on the lyophilization of CDNs in the absence of lyoprotectant. We observed that the mean hydrodynamic diameter of CDNs increased significantly (334 ± 11.1 nm) and that the protein content was reduced by 15.9%, clearly indicating that the combination of lyoprotectant and non-ionic surfactant is essential to achieve the observed protective effects during lyophilization.

### 3.3. Impact of Lyoprotectant Concentration on the Lyophilization of CDNs

As a next step, we investigated the influence of lyoprotectant concentrations at three different lyoprotectant/CDNs’ protein ratios (50:1, 100:1, and 200:1) in the presence of polysorbate 80 (0.01% *w*/*v*) (Table 1). Compared to the freshly prepared CDNs (before lyophilization), both sucrose and trehalose in the previously used ratio of 100:1:0.2 (lyoprotectant:CDNs protein:polysorbate 80) preserved the stability of CDNs, as evidenced by comparable hydrodynamic diameter and zeta potential. After lowering the concentration of each lyoprotectant (lyoprotectant:CDNs protein:polysorbate 80 ratio being 50:1:0.2), the protective effect on the size was strongly reduced (Table 1). It could be inferred that those lower concentrations of the lyoprotectant are not sufficient to protect the vesicles and proteins from destabilization during the freeze-drying. The higher concentration of lyoprotectant tested (lyoprotectant:CDNs protein:polysorbate 80 ratio 200:1:0.2) also yielded a lower degree of lyoprotection (Table 1). This phenomenon could be due to the creation of enhanced transmembrane osmotic stress known to occur at high concentrations of sugars, and consequently aggregation of nanovesicles. These findings suggest that too low and too high concentrations of sugars could diminish the degree of lyoprotection. Among the factors responsible for vesicle agglomeration upon reconstitution, the surface charge is also considered one of the prime contributors; hence, the zeta potential was measured before and after lyophilization of CDNs. As expected, in view of the type of lyoprotectant and non-ionic surfactant used, we did not observe any significant change in surface charge of the CDNs after lyophilization (Table 1).

Based on these findings, we considered the ratio 100:1:0.2 of lyoprotectant (sucrose or trehalose):CDNs’ protein:polysorbate 80 as optimal for lyophilization of CDNs. These formulations were indicated as “sucrose-lyoprotected CDNs” (S1) and “trehalose-lyoprotected” CDNs (T1), respectively, while CDNs lyophilized without lyoprotectant were referred to as “non-lyoprotected CDNs” (W) (Table 1).

Moreover, to better understand the effect of mechanical stress on the biomolecular structure and biological activities of CDNs, a heat-denatured sample (negative control) was prepared by heating CDNs at 90 ºC for 30 min. This sample was indicated as “heated CDNs” (H). As a result of heating, the mean hydrodynamic diameter and protein concentration were observed to be around 680 nm and 371.77 µg/mL, respectively, indicating a significant (*p* < 0.001) increment in size and reduction in protein concentration (by 36.5%) after the heating process (Table 1, H). This heated dispersion was used as a control to assess whether the observed lyophilization-induced changes regarding vesicle size and decrease in protein content of CDNs are associated with lower cellular interaction and compromised biological activity.

Under transmission electron microscopy, CDNs lyophilized in the presence of lyoprotectant and polysorbate 80 appeared as well-preserved, spherical nanovesicles, suggesting that the morphology remained unaffected by the process (Supplementary Figure S1A). In addition, through field emission scanning electron microscopy, images of the same CDN samples showed an amorphous and porous cake (Supplementary Figure S1B).

Noteworthy, we did not observe any significant change in the concentration of CDNs (i.e., number of vesicles per mL) induced by lyophilization for sucrose-lyoprotected CDNs (S1) and trehalose-lyoprotected CDNs (T1) samples in comparison with fresh samples, indicating that CDNs’ integrity was maintained through the process (Supplementary Figure S4).

### 3.4. Residual Moisture Content and Glass Transition Temperature (Tg) of Lyophilized CDNs

Low residual moisture content is highly desirable for lyophilized products. Water molecules can act as a plasticizer and decrease the Tg of the lyophilized product. In addition, high residual moisture accelerates microbial growth and promotes the agglomeration of particles, and increases chemical molecular instability due to deamidation and oxidation reactions during the time of storage [28].

**Figure 1:**
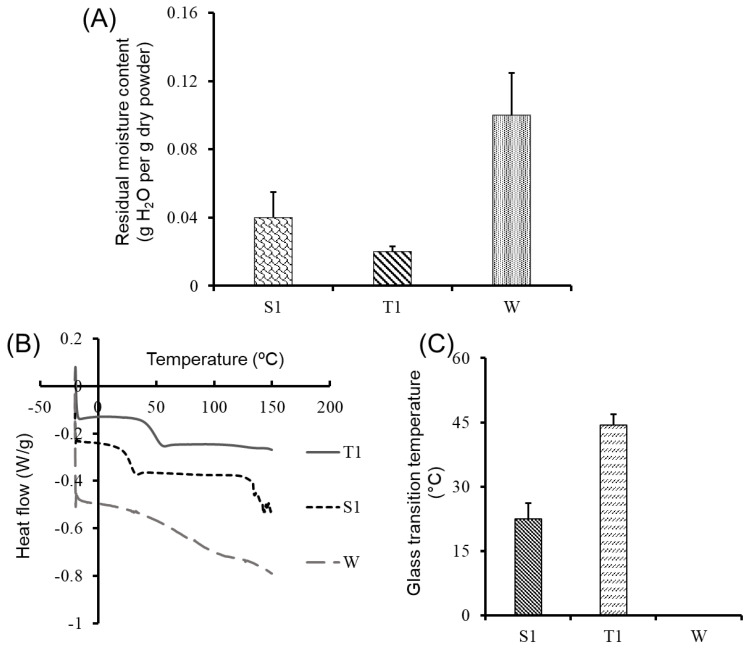
(**A**) Residual moisture content (g H_2_O per g of dry powder) of lyophilized samples measured directly after lyophilization by gravimetric analysis. (**B**) Glass transition temperature (Tg) of lyophilized CDNs with sucrose (S1) or trehalose (T1) as lyoprotectant and without lyoprotectant (W), measured by DSC. (**C**) Tg of sucrose-lyoprotected CDNs (S1), trehalose-lyoprotected CDNs (T1), and non-lyoprotected CDNs (W) as determined by DSC. The W sample did not show any Tg value. Data presented are mean ± SD from three independent experiments (*n* = 3).

The non-lyoprotected freeze-dried CDN powder (W) was characterized by a higher residual moisture content (0.1 ± 0.025 g H_2_O per g of dry powder) as compared to sucrose-lyoprotected CDNs (S1) (0.04 ± 0.015 g H_2_O per g dry powder) and trehalose-lyoprotected CDNs (T1) (0.02 ± 0.003 g H_2_O per g dry powder) (Figure 1A). Residual moisture content in the sucrose- and trehalose-lyoprotected CDNs was within an acceptable range for lyophilized biological products, i.e., less than 0.05 g H_2_O per g dry power [18]. We speculate that non-lyoprotected CDNs powders hold more water molecules in between denatured proteins, resulting in higher residual moisture content. Sucrose-lyoprotected CDNs (S1) showed higher residual moisture content than trehalose-lyoprotected CDNs (T1) (Figure 1A), possibly due to the higher hygroscopic nature of sucrose as compared to trehalose [20]. Similar observations were reported for lyophilization of liposomes using sucrose and trehalose as lyoprotectants [20].

Besides residual moisture content, Tg is also considered as one of the most important parameters in determining the storage stability of lyophilized biological products [18]. In lyophilized products with storage temperature exceeding the Tg value, a second-order transition can occur from a rigid solid state to a viscoelastic rubbery state, usually leading to the collapse of the product [46]. It has been reported that the Tg value inversely correlates with the residual moisture content [18]. Accordingly, while sucrose-lyoprotected CDNs (S1) showed higher residual moisture than trehalose-lyoprotected CDNs (T1), T1 showed higher Tg than S1 (Figure 1C). Given the limitations of this gravimetric analysis, future studies will include a Karl–Fisher method to confirm the residual moisture. Nonetheless, as determined by DSC, Tg of the lyophilized CDNs had distinct signals in the sucrose-lyoprotected CDN (S1) and trehalose-lyoprotected CDN powders (T1), while no signal was detectable in the non-lyoprotected CDN powder (W) (Figure 1B, C). As we observed a higher Tg value for trehalose-lyoprotected CDNs (T1, 44.42 ± 2.48 ºC) as compared to sucrose-lyoprotected CDNs (S1, 22.42 ± 3.69 ºC), we can expect that CDNs lyophilized with trehalose show better storage stability than those lyophilized with sucrose. Taken together, the demonstrated low residual moisture content and sufficiently high Tg values point to the formation of an amorphous glassy matrix (as a confirmation of the vitrification hypothesis, Supplementary Figure S2) during lyophilization of CDNs occurring with both lyoprotectants. These findings represent favorable formulation attributes, potentially enabling the long-term solid-state storage of the lyophilized products below their Tg values.

### 3.5. Protein Content and Secondary Structure

**Figure 2:**
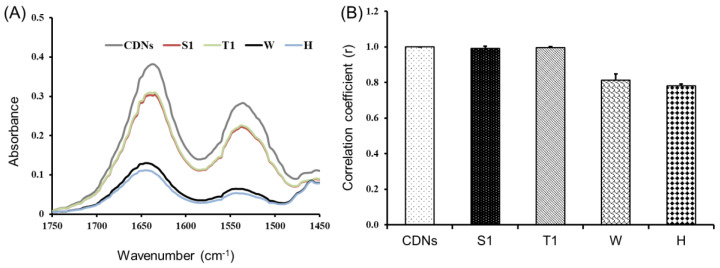
Characterization of the overall secondary structure of proteins in CDNs. (**A**) ATR-FTIR spectra of freshly prepared CDNs, lyophilized CDNs with either sucrose (S1) or trehalose (T1) as lyoprotectants, non-lyoprotected CDNs (W), and heat-denatured CDNs (H) in the 1750–1450 cm^−1^ spectral region. (**B**) Correlation coefficient (r) values comparing ATR-FTIR spectra of freshly prepared CDNs and the different CDN lyophilization groups. Data represent mean ± SD determined from three independent experiments (*n* = 3).

ATR-FTIR analysis was employed to determine the overall secondary structure of the proteins present in both freshly prepared CDNs and lyophilized CDNs. This method was reported for the characterization of multiple proteins in cell membranes [18]. Figure 2A shows the amide-I and amide-II bands at 1635 and 1530 cm^−1^, respectively. Amide-I and amide-II are two major absorbance bands of proteins in the infrared region: amide-I is mainly associated with C=O stretching vibration and is directly associated with the backbone conformation of proteins, whereas amide-II results from N-H bending vibration and from C-N stretching vibration [33]. Heated (H) and non-lyoprotected (W) CDNs showed a significant reduction in the absorbance profiles in FTIR spectra as compared with freshly prepared CDNs, sucrose-lyoprotected CDNs (S1), and trehalose-lyoprotected CDNs (T1), pointing to denaturation of the proteins present in these CDN. Sucrose-lyoprotected CDNs and trehalose-lyoprotected CDNs showed no sign of denaturation at amide-I and amide-II spectral region, indicating the protective nature of sugars during lyophilization of CDNs.

To reveal more clearly eventual differences among different samples, a correlation coefficient (r) was calculated and compared with CDNs (Figure 2B). The correlation coefficient of freshly prepared CDNs was retained to be 1 and, once lyoprotected with sucrose (S1) and trehalose (T1), the r-value was still optimal (r = 0.992 and 0.995, respectively), suggesting that FTIR spectra of S1 and T1 highly correlated with FTIR spectra of fresh CDNs. On the other hand, the r values for non-lyoprotected CDNs (W) and heated CDNs (H) were determined to be 0.814 and 0.782, respectively. This indicates that the surface membrane proteins of the CDNs were at least partially denatured during lyophilization without lyoprotectant (W) and during heating (H). Our results are in agreement with the study reported by Zhang et al. on lyophilization of mammalian cells using trehalose as a lyoprotectant [18].

We also determined the proteins’ secondary structure and contents of freshly prepared as well as lyophilized CDNs using a Far-UV CD spectrophotometer. The results from far-UV CD analysis strongly corroborate the data from ATR-FTIR spectroscopy. Sucrose-lyoprotected CDNs and trehalose-lyoprotected CDNs showed similar CD intensity when compared with freshly prepared CDNs. However, heated CDNs and non-lyoprotected CDNs showed significantly lower CD intensity as compared with CDNs and lyoprotected samples, suggesting that the proteins of CDNs were at least partly denatured during heating and lyophilization without lyoprotectant (Supplementary Figure S5). Both the FTIR and CD findings prove that lyoprotectants could protect the overall secondary structure of the proteins. CDNs are derived from cells and are expected to retain their cell membrane topology during lyophilization to preserve their structure and biological activity. The structural perturbations induced by heat denaturation and lyophilization without lyoprotectants may alter these key features of CDNs. Taken together, our results indicate that sucrose- and trehalose-lyoprotected CDNs likely have similar structural and biological activity characteristics such as fresh CDNs.

### 3.6. Cellular Association Study

The intrinsic targeting capability of U937 monocytic cells towards inflammatory and tumor sites has been well documented [2,29,31]. This phenomenon seems attributable to certain proteins present on the surface of U937 cells, which act as recognition sites between these U937 immune cells and inflamed/cancerous tissue [47]. Accordingly, we previously reported that CDNs derived from U937 cells were preferentially associated with cancer cells (HeLa) over healthy cells (HEK 293) in a co-culture model [30]. In another study by Jang et al., the cellular association of U937-derived CDNs with human umbilical vein endothelial cells was markedly reduced when “counter receptors” present on the surface of CDNs were removed via trypsinization [2]. The group of Krishnamurthy et al. showed that the cellular association of PLGA nanoparticles with MCF-7 breast cancer cells was enhanced by coating them with U937 monocytic cell membranes [31]. These findings point to the intrinsic targeting ability of U397-CDNs by virtue of inherited membranes from their parent U937 monocytic cells [31].

In this study, we demonstrated that CDNs were taken up intracellularly and were not simply confined/adsorbed onto the cellular plasma membranes through confocal laser scanning microscopy analysis (Supplementary Figure S6), which was performed on fluorescently labeled U937 cell-derived CDNs, sucrose-lyoprotected CDNs (S1), trehalose-lyoprotected CDNs (T1), non-lyoprotected CDNs (W), and heat-denatured CDNs (H) incubated with cells at different time points. The purpose of this experiment was to assess the impact of the lyophilization process on the cellular association of CDNs mediated by their surface proteins. Towards this purpose, HeLa and CT26 cells were selected because the intrinsic targeting capability of CDNs derived from U937 monocytic cells to these cancer cells has been demonstrated and compared with conventional nanovesicular systems such as liposomes [29]. Moreover, BMDM was selected to assess the cellular association of CDNs with primary immune cells as an additional control for non-cancerous and non-immortalized cell lines.

**Figure 3:**
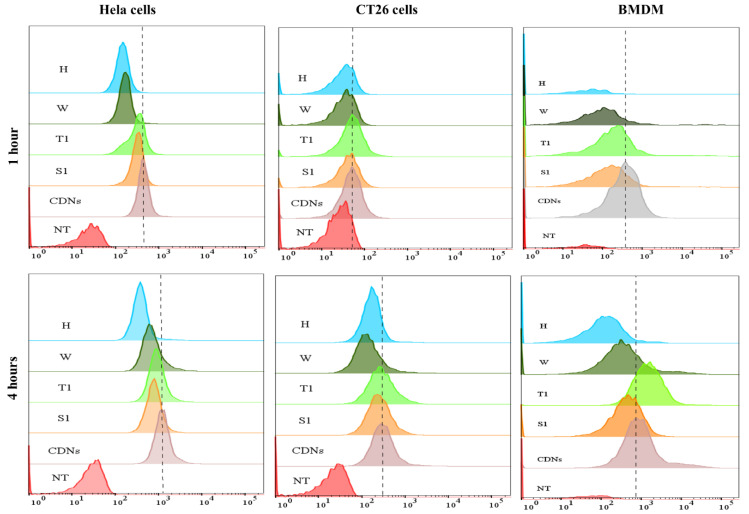
Cellular association of CDNs. Cy3-labeled samples were incubated with HeLa cells, CT26 cells or BMDM for 1 h or 4 h, and then analyzed by flow cytometry and compared between freshly prepared CDNs, sucrose-lyoprotected CDNs (S1), trehalose-lyoprotected CDNs (T1), non-lyoprotected CDNs (W), and heat-denatured CDNs (H). *X*-axis represents log fluorescence intensity of Cy3, and *Y*-axis represents counts, NT = non-treated cells (images are representations from three independent experiments).

As a confirmation of these results, cellular association of Cy3-labeled CDNs [11] by flow cytometry showed the increment in the number of Cy3-positive cells over time in all tested cell lines, indicating a time-dependent association of CDNs with cells (Figure 3).

In the case of HeLa cells, we observed a 2.6- and 2.9-fold higher association of fresh CDNs when compared with non-lyoprotected CDNs (W) and heated CDNs (H), respectively, after 1 h incubation. Similar results were obtained after 4 h incubation, with 1.8-fold and 3.3-fold higher association of fresh CDNs as compared to non-lyoprotected CDNs (W) and heated CDNs (H), respectively. In contrast, no significant change was shown for the cellular association of sucrose-lyoprotected CDNs (S1) and trehalose-lyoprotected CDNs (T1) compared with fresh CDNs at both time points.

In the case of CT26 cells, after 1 h incubation, we did not observe much difference in the profile between the various CDN types, whereas after 4 h incubation fresh CDNs showed 2.2- and 1.7-fold higher association than non-lyoprotected CDNs (W) and heated CDNs (H), respectively, and no significant difference with sucrose-lyoprotected CDNs (S1) and trehalose-lyoprotected CDNs (T1).

In the case of BMDM cells, we observed a 3.5- and 9.8-fold higher association of fresh CDNs when compared with non-lyoprotected CDNs (W) and heated CDNs (H), respectively, after 1 h incubation. Similarly, 2.1- and 2.7-fold higher association of fresh CDNs were observed as compared with non-lyoprotected CDNs (W) and heated CDNs (H), respectively, after 4 h of incubation. In line with the cancer cells results, no significant change was shown for sucrose-lyoprotected CDNs (S1) and trehalose-lyoprotected CDNs (T1) compared with fresh CDNs at 1 h incubation. However, remarkably, we observed a higher association of only the trehalose-lyoprotected CDNs (T1) by BMDM after 4 h incubation compared to fresh CDNs. One possible explanation for the latter finding is that trehalose binds to the C-type lectin (Mincle) receptor expressed on BMDM cells and that may have stimulated cellular uptake [48,49]. Nonetheless, the results obtained with the BMDM cells also confirm that lyophilization with a suitable lyoprotectant can preserve the interaction of CDNs with target cells.

### 3.7. Antioxidant Capacity

**Figure 4:**
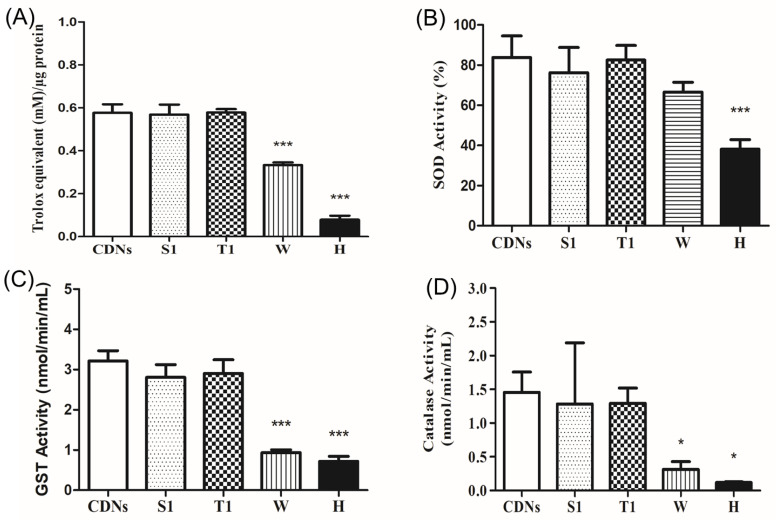
Antioxidant capacity of fresh CDNs and lyophilized CDNs. (**A**) Total antioxidant capacity was expressed as Trolox equivalent (mM)/µg of protein. (**B**–**D**) Antioxidant activities of SOD (**B**), GST (**C**), and catalase (**D**) were measured for fresh CDNs and lyophilized CDNs. * *p* < 0.05, *** *p* < 0.001, compared with fresh CDNs. Data represent mean ± SD (*n* = 3).

As U937 monocytic cell-derived exosomes were reported to carry several antioxidant enzymes (AOEs, namely SOD1, SOD2, catalase, GSTK1, and PRDX6) inherited from parent cells [12], we examined if lyophilization affected the antioxidant capacity of CDNs due to the presence of those antioxidant enzymes derived from U937 cells. Figure 4A shows that lyophilization of CDNs in the presence of sucrose (S1) and trehalose (T1) well preserved the total antioxidant capacity of the CDNs when compared with fresh CDNs (*p* > 0.05). Lyophilization of CDNs without lyoprotectant (W) and heat-denatured CDNs (H), however, showed a significant reduction (*p* < 0.001) of the total antioxidant capacity as compared with fresh CDNs. AOEs (SOD, GST, and catalase) of the CDNs were preserved during lyophilization in the presence of lyoprotectant sucrose (S1) and trehalose (T1). As expected, the SOD activity was significantly (*p* < 0.001) reduced in heat-denatured CDNs (H) compared with fresh CDNs (Figure 4B). SOD, GST, and catalase activity were significantly reduced in case of non-lyoprotected CDNs (W) and heat-denatured CDNs (H) (Figure 4C,D), with the exception of SOD activity in the case of non-lyoprotected CDNs (W). Heat denaturation treatment of CDNs (H) exerted a more pronounced detrimental effect on the antioxidant capacity. These results show that the stress generated during lyophilization does not necessarily compromise the activity of the antioxidant enzymes when a proper lyoprotectant is used, combined with polysorbate 80.

### 3.8. Long-Term Stability of Lyophilized CDNs

**Figure 5:**
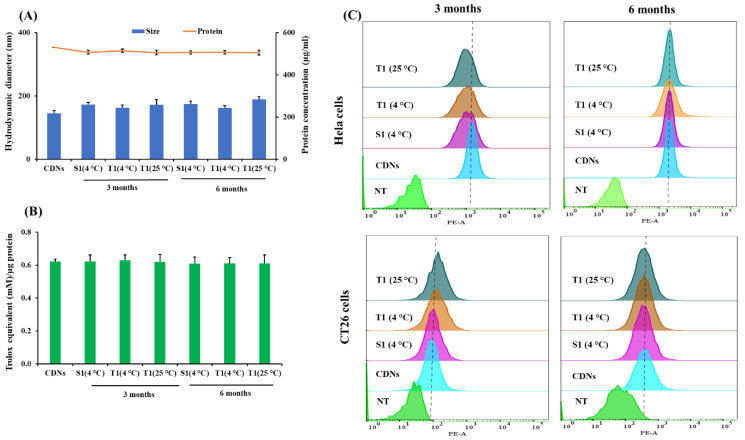
Long-term stability of the lyophilized CDNs after 3 and 6 months stored at 4 °C (for sucrose-lyoprotected CDNs (S1)) and 4 and 25 °C (for trehalose-lyoprotected CDNs (T1)). (**A**) Hydrodynamic diameter and protein concentration were measured by Zetasizer nano and BCA assay, respectively. (**B**) Total antioxidant capacity was measured by TAC assay. (**C**) Cellular association of Cy3-labeled samples with HeLa and CT26 cells after incubation for 4 h was analyzed by flow cytometry. Data are expressed as mean ± SD of three independent experiments (*n* = 3).

Sucrose- and trehalose-lyophilized CDNs were evaluated for their long-term stability regarding changes in size, total protein content, and total antioxidant capacity at two different storage temperatures, i.e., 4 and 25 °C. Based on their Tg, sucrose-lyoprotected CDNs were stored at 4 °C (S1, Tg 22 °C) while trehalose-lyoprotected CDNs were stored at 4 and 25 °C (T1, Tg 44 °C), for 3 and 6 months. The changes in hydrodynamic diameter, total protein contents, and total antioxidant capacity of CDNs were insignificant over a period of 6 months as presented in Figure 5A,B. Moreover, upon reconstitution, the freeze-dried preparations stored for 6 months exhibited a comparable cellular association profile in HeLa and CT26 cell cultures than freshly prepared CDNs (Figure 5C). This indicates that an effective stabilization of the surface was achieved and maintained for at least 6 months. Taken together, these results provide considerable evidence that our CDNs lyophilized in the presence of lyoprotectants (sucrose or trehalose) and polysorbate 80 can be stored over a long time in a dried state without losing vesicle structure and membrane topology. Furthermore, our results suggest that a cold chain storage condition is not required for trehalose-lyoprotected CDNs (T1). Our lyophilization protocol may be applied to other cell-derived vesicles to achieve long-term storage in dry powder form at room temperature.

Based on the Tg value and long-term stability study outcome (at 25 °C), trehalose-lyoprotected CDNs (T1) were considered for further in vivo studies.

### 3.9. Preservation of Cardioprotective Effect of CDNs after Lyophilization

**Figure 6:**
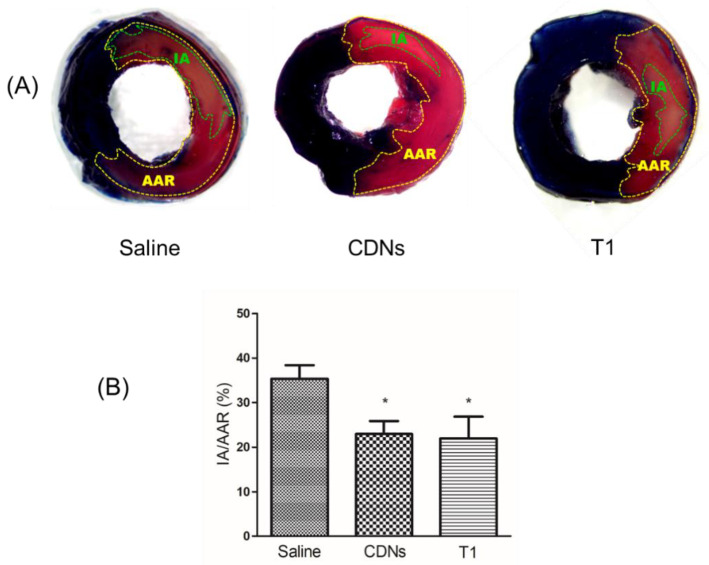
Cardioprotective effect of CDNs and trehalose-lyoprotected CDNs (T1). CDNs or saline were injected to the mice via tail vein 5 min before reperfusion and the infarct size was measured 24 h after reperfusion. (**A**) Representative images indicating IA and AAR for saline control, fresh CDNs, and trehalose-lyoprotected CDN (T1)-treated mice after TTC staining. (**B**) Infarct size was expressed as IA/AAR (%). Data represent mean ± SEM, *n* = 8–10 mice per group, * *p* < 0.05 compared with saline control. IA, infarcted area, dead heart tissue in pale; AAR, area at risk, the heart tissue not stained in blue.

Ischemia/reperfusion (I/R) injury, observed during revascularization treatment of acute myocardial infarction, is defined as an acute interruption in blood flow with subsequent restoration of perfusion, leading to cell death and functional damage of the cardiomyocytes in that infarcted myocardium [50]. Currently, exosomes of various cell origins have shown cardioprotective effects against I/R injury [34,51,52]. Given that U937 monocytic CDNs mimic U937-derived exosomes regarding proteins, lipids, and enzymes [11], we hypothesized that U937 monocytic CDNs, similarly to exosomes, could display cardioprotective effects. To test this hypothesis, for the first time reported, we examined if intravenously administered freshly prepared CDNs could decrease the tissue necrotic area within the infarcted myocardium, i.e., infarct size as indicated in Figure 6A, in a mouse model of myocardial I/R injury. As shown in Figure 6B, freshly prepared CDNs from U937 monocytic cells showed a significant reduction (*p* < 0.05) in myocardial infarct size (22.94 ± 2.91%) within 24 h after administration of CDNs, as evidenced by the smaller percentage of infarct area with respect to the area at risk in CDNs-treated heart than that in saline control heart (35.29 ± 3.07%). Interestingly, lyophilized CDNs with trehalose (T1) and polysorbate 80 reduced the infarct size (21.94 ± 4.90%) to the same extent as freshly prepared CDNs. These results suggest that CDNs, similar to exosomes [34], could exert cardioprotective effects and that lyophilization of CDNs with trehalose did not compromise this therapeutic activity.

## 4. Conclusions

In conclusion, lyoprotectants sucrose and trehalose, in combination with polysorbate 80, preserved the physicochemical properties, surface proteins, vesicular integrity, and biological activities of CDNs regarding cellular interaction during lyophilization. We have developed an optimal lyoprotectant formulation for lyophilization of CDNs with a ratio 100:1:0.2 of trehalose:CDNs protein:polysorbate 80. Lyophilized CDNs with trehalose can be conveniently stored at room temperature for a long period of time (>6 months), easily reconstituted, and used whenever desired.

## Figures and Tables

**Table 1:** Effect of lyoprotectant type and amount on physicochemical properties of CDNs after lyophilization. Hydrodynamic diameter, zeta potential, and protein concentration of lyophilized CDNs were determined after reconstitution. Results shown are mean ± SD of three independent experiments. ^a^ *p* < 0.001, ^b^ *p* < 0.01, ^c^ *p* < 0.001 compared with fresh CDNs before lyophilization.

Lyophilization Conditions for CDNs	Hydrodynamic Diameter (nm)	Zeta Potential (mV)	Protein Concentration (µg/mL)	% Protein Loss
CDNs before lyophilization	124.80 ± 8.43	−7.04 ± 0.8	538.92 ± 6.12	----
Non-lyoprotected CDNs (W)	394.93 ± 30.92 ^a^	−8.45 ± 2.5	460.03 ± 4.49 ^c^	14.6
Sucrose:CDNs protein (weight) (100:1)	330.27 ± 27.27 ^a^	−8.28 ± 1.5	503.37 ± 6.32 ^c^	7.7
Sucrose:CDNs protein:polysorbate 80 (50:1:0.2)	212.93 ± 24.60 ^a^	−6.67 ± 1.2	510.34 ± 10.69 ^c^	5.6
Sucrose:CDNs protein:polysorbate 80 (100:1:0.2) (S1)	147.77 ± 10.14	−7.86 ± 2.9	518.55 ± 2.94 ^b^	4.0
Sucrose:CDNs protein:polysorbate 80 (200:1:0.2)	252.83 ± 5.93 ^a^	−9.36 ± 1.7	494.85 ± 2.80 ^c^	8.5
Trehalose:CDNs protein (100:1)	316.10 ± 11.12 ^a^	−7.82 ± 1.6	508.92 ± 7.40 ^c^	6.0
Trehalose:CDNs protein:polysorbate 80 (50:1:0.2)	220.80 ± 13.52 ^a^	−7.04 ± 2.1	507.70 ± 2.26 ^c^	6.5
Trehalose:CDNs protein:polysorbate 80 (100:1:0.2) (T1)	143.9 ± 5.39	−8.25 ± 2.0	528.45 ± 3.18	2.0
Trehalose:CDNs protein:polysorbate 80 (200:1:0.2)	226.10 ± 9.01 ^a^	−6.68 ± 2.2	506.73 ± 3.53 ^c^	6.1
Heated CDNs (H)	680.43 ± 35.66 ^a^	−10.9 ±2.3	371.77 ± 3.87 ^c^	36.5

## Data Availability

The data presented in this study are available on request from the corresponding authors.

## Supplementary Materials

The following are available online at https://www.mdpi.com/article/10.3390/pharmaceutics13071052/s1, Figure S1. Microscopic analysis of CDNs. (A) TEM images of CDNs before lyophilization (top left), sucrose-lyoprotected CDNs (S1) and trehalose-lyoprotected CDNs (T1), respectively. Scale bar 100 nm. (B) FESEM images of sucrose-lyoprotected CDNs (S1), trehalose-lyoprotected CDNs (T1) and non-lyoprotected CDNs (W), scale bar 1 µm. Figure S2. Schematic representation of vitrification hypothesis during lyophilization, in which protein is embedded into lyoprotectant resulting in the formation of an amorphous highly viscous glassy matrix characterized by a specific glass transition temperature (Tg). Figure S3. Schematic representation of water replacement hypothesis during lyophilization and rehydration of CDNs. (A) During hydrated state, lipid bilayers of the CDNs are loosely packed due to presence of the embedded water molecules in the bilayers. (B) Lyophilization without lyoprotectant, where lipid bilayers display packing defects during freezing and drying. (C) Upon rehydration, bilayers become leaky and membrane proteins present in the surface are degraded. (D) Lyophilization with lyoprotectant, in which the water molecules are eventually replaced with lyoprotectant molecules that reduce the van der Waals interactions between the lipid bilayers in the dry state and maintain the lipid bilayer packing intact. (E) Upon rehydration, bilayers packing is well protected as well as surface membrane proteins. Figure S4. Concentration of nanovesicles in freshly prepared CDNs, lyophilized CDNs with sucrose (S1) and trehalose (T1), lyophilized CDNs without lyoprotectant (W) and heat-denatured CDNs (H) measured via nanoparticle tracking analysis (NTA). Data represent mean ± SD, *n* = 3. Figure S5. Characterization of secondary structure and composition of proteins in CDNs. (A) Far-UV CD spectral analysis of freshly prepared CDNs, lyophilized CDNs with sucrose (S1) and trehalose (T1), lyophilized CDNs without lyoprotectant (W) and heat-denatured CDNs (H) within 190–260 nm wavelength. (B) Quantification of different sec-ondary structures of proteins measured by far-UV CD spectral analysis. Data represent mean value ± SD determined from three independent experiments (*n* = 3). Figure S6. Cellular uptake analysed by confocal microscopy. Cy3 labelled samples of freshly prepared CDNs, lyophilized CDNs with sucrose (S1) and trehalose (T1), lyophilized CDNs without lyoprotectant (W) and heat-denatured CDNs (H) were incubated with HeLa cells for 6 hours. Nuclei and cell membrane of HeLa cells were stained with Hoechst 33342 dye and Cellmask green, respectively. Scale bars represent 20 µm.
