# Supplementary material for: Lyophilization Preserves the Intrinsic Cardioprotective Activity of Bioinspired Cell-Derived Nanovesicles
Source: Pharmaceutics. 2021 Jul 9;13(7):1052. doi: 10.3390/pharmaceutics13071052 (PMC8309024; doi:10.3390/pharmaceutics13071052)
Supplement: Supplementary file 1 [file pharmaceutics-13-01052-s001.zip › pharmaceutics-1267459-supplementary.pdf]

pharmaceutics

MDPI

Supplementary Materials: Lyophilization Preserves the Intrinsic Cardioprotective Activity of Bioinspired Cell-Derived Nanovesicles

Yub Raj Neupane, Chenyuan Huang, Xiaoyuan Wang, Wei Heng Chng, Gopalakrishnan Venkatesan, Olga Zharkova, Matthias Gerhard Wacker, Bertrand Czarny, Gerrit Storm, Jiong-Wei Wang and Giorgia Pastorin

Microscopic evaluation

Vesicular morphology of CDNs was examined under Transmission Electron Microscopy (TEM). Samples were fixed with 2.5% glutaraldehyde for 1 hour at 4 °C, 20 μL sample was deposited onto a Formvar Film 200 mesh (CU, FF200-Cu grid) for 15 minutes and stained with 5% gadolinium triacetate for 1 minute. The samples were visualized using liquid TEM (FEI TECNAI SPIRIT G2 with ICORR).

The microstructure and texture of lyophilized CDNs were examined by Field Emission Scanning Electron Microscopy (FESEM). Samples were fixed on aluminium stubs and coated with Pt\Pd in an auto fine coater (JFC-1600, JEOL). Images were recorded with JEOL field emission scanning electron microscope at an operating voltage of 5 kV (JSM-6700F).

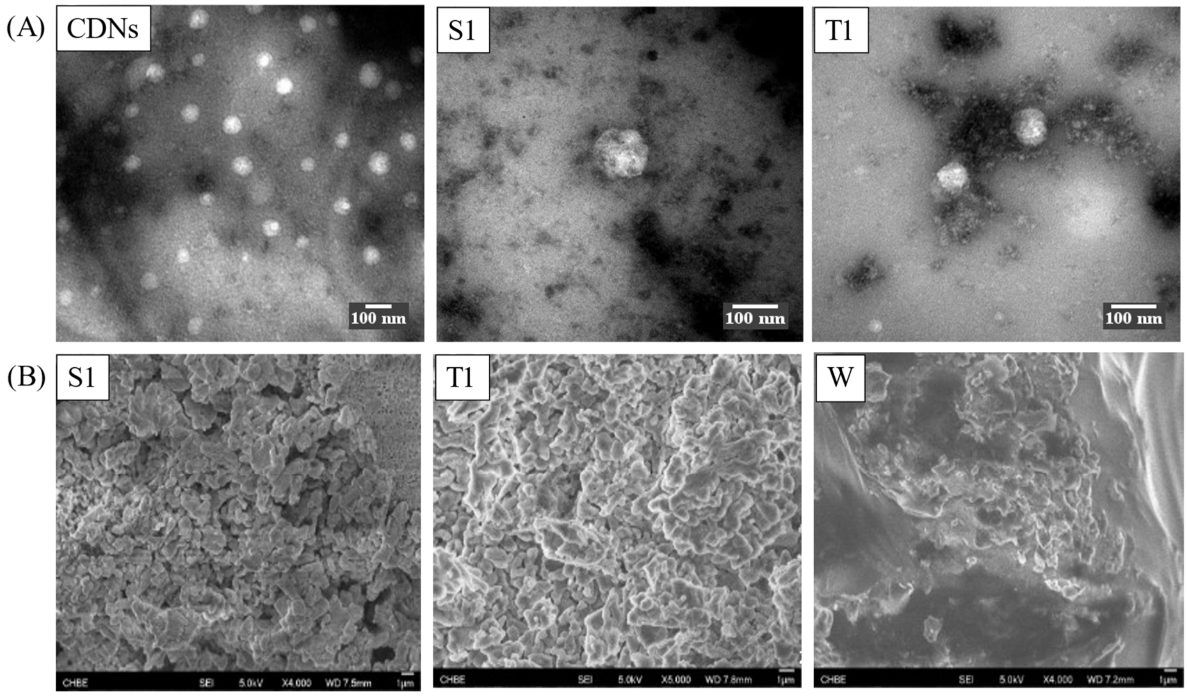

Figure S1. Microscopic analysis of CDNs. (**A**) TEM images of CDNs before lyophilization (top left), sucrose-lyoprotected CDNs (S1) and trehalose-lyoprotected CDNs (T1), respectively. Scale bar 100 nm. (**B**) FESEM images of sucrose-lyoprotected CDNs (S1), trehalose-lyoprotected CDNs (T1) and non-lyoprotected CDNs (W), scale bar 1 μm.

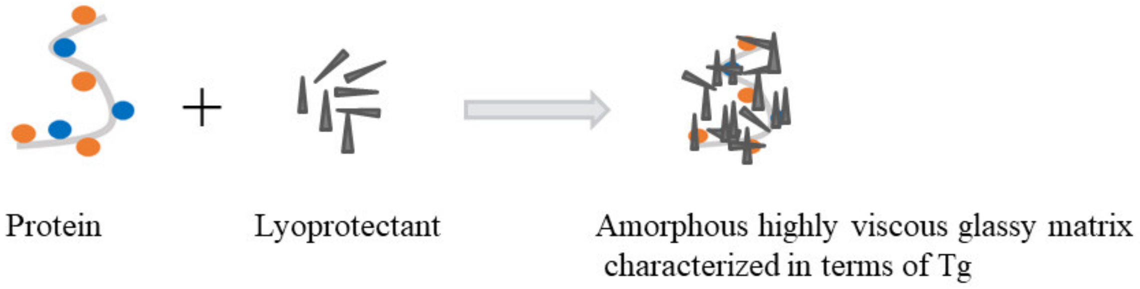

Figure S2. Schematic representation of vitrification hypothesis during lyophilization, in which protein is embedded into lyoprotectant resulting in the formation of an amorphous highly viscous glassy matrix characterized by a specific glass transition temperature (Tg).

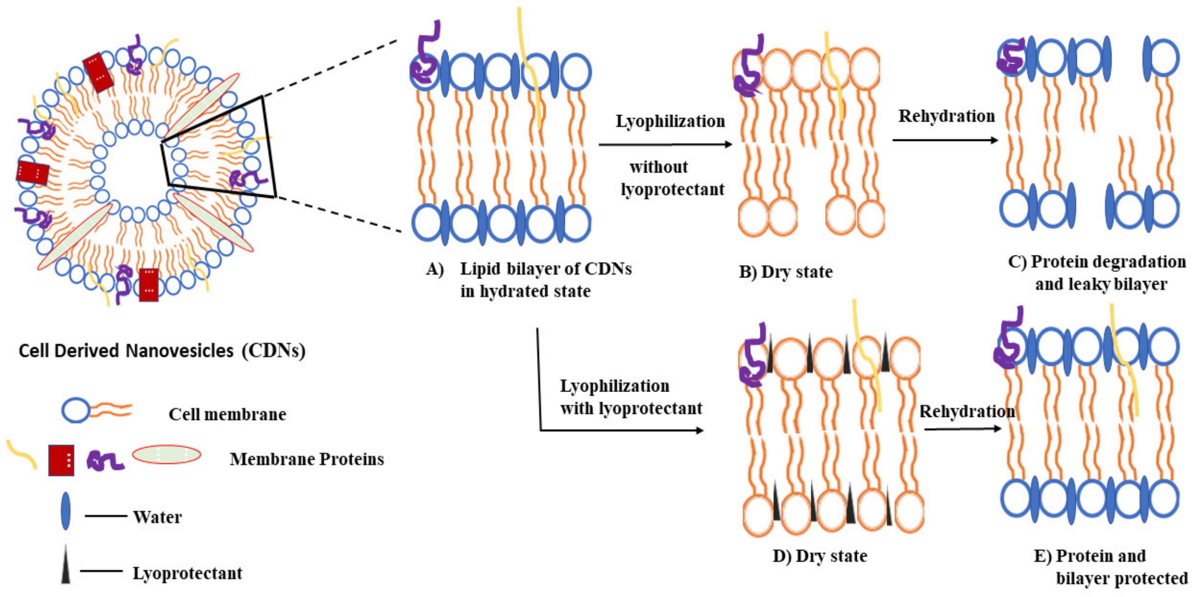

Figure S3. Schematic representation of water replacement hypothesis during lyophilization and rehydration of CDNs. (**A**) During hydrated state, lipid bilayers of the CDNs are loosely packed due to presence of the embedded water molecules in the bilayers. (**B**) Lyophilization without lyoprotectant, where lipid bilayers display packing defects during freezing and drying. (**C**) Upon rehydration, bilayers become leaky and membrane proteins present in the surface are degraded. (**D**) Lyophilization with lyoprotectant, in which the water molecules are eventually replaced with lyoprotectant molecules that reduce the van der waals interactions between the lipid bilayers in the dry state and maintain the lipid bilayer packing intact. (**E**) Upon rehydration, bilayers packing is well protected as well as surface membrane proteins.

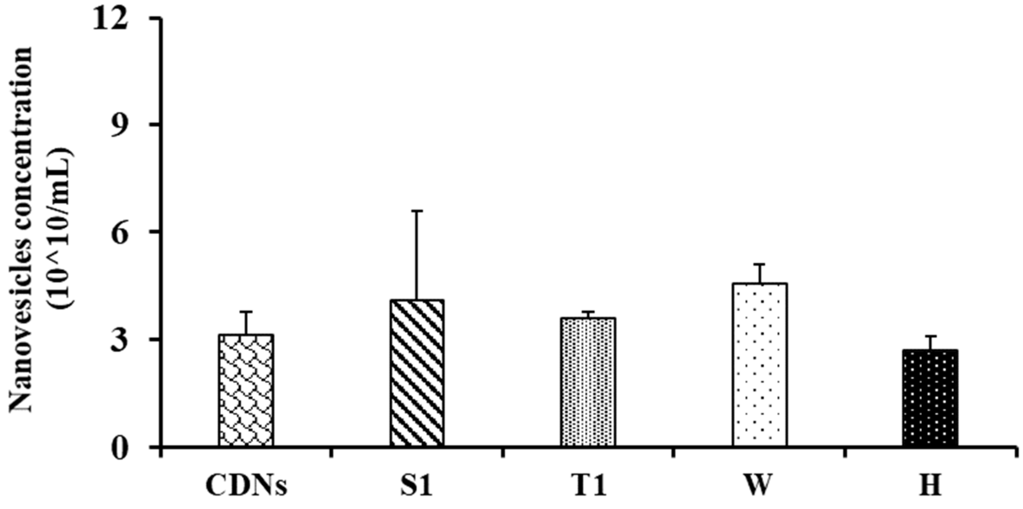

Figure S4. Concentration of nanovesicles in freshly prepared CDNs, lyophilized CDNs with sucrose (S1) and trehalose (T1), lyophilized CDNs without lyoprotectant (W) and heat-denatured CDNs (H) measured *via* Nanoparticle Tracking Analysis (NTA). Data represent mean ± SD, $n = 3$.

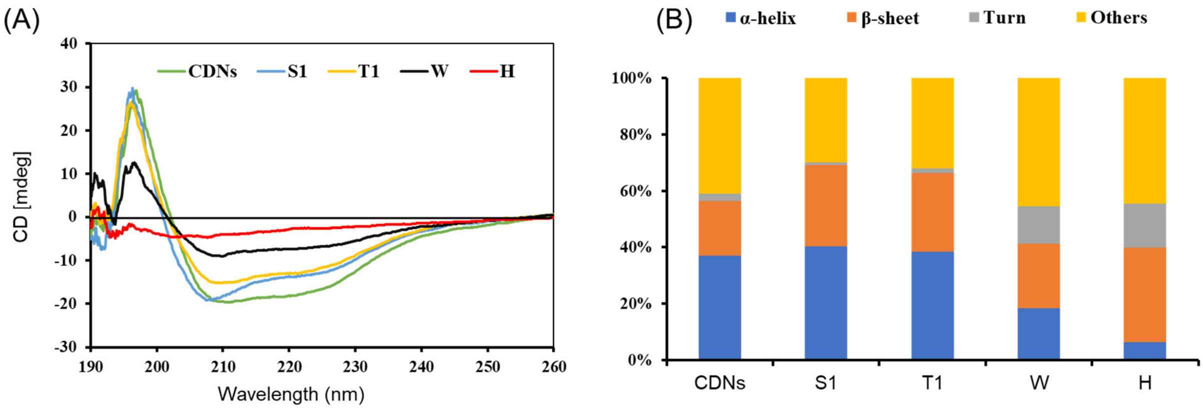

Figure S5. Characterization of secondary structure and composition of proteins in CDNs. (A) Far-UV CD spectral analysis of freshly prepared CDNs, lyophilized CDNs with sucrose (S1) and trehalose (T1), lyophilized CDNs without lyoprotectant (W) and heat-denatured CDNs (H) within 190-260 nm wavelength. (B) Quantification of different secondary structures of proteins measured by Far-UV CD spectral analysis. Data represent mean value ± SD determined from three independent experiments ($n = 3$).

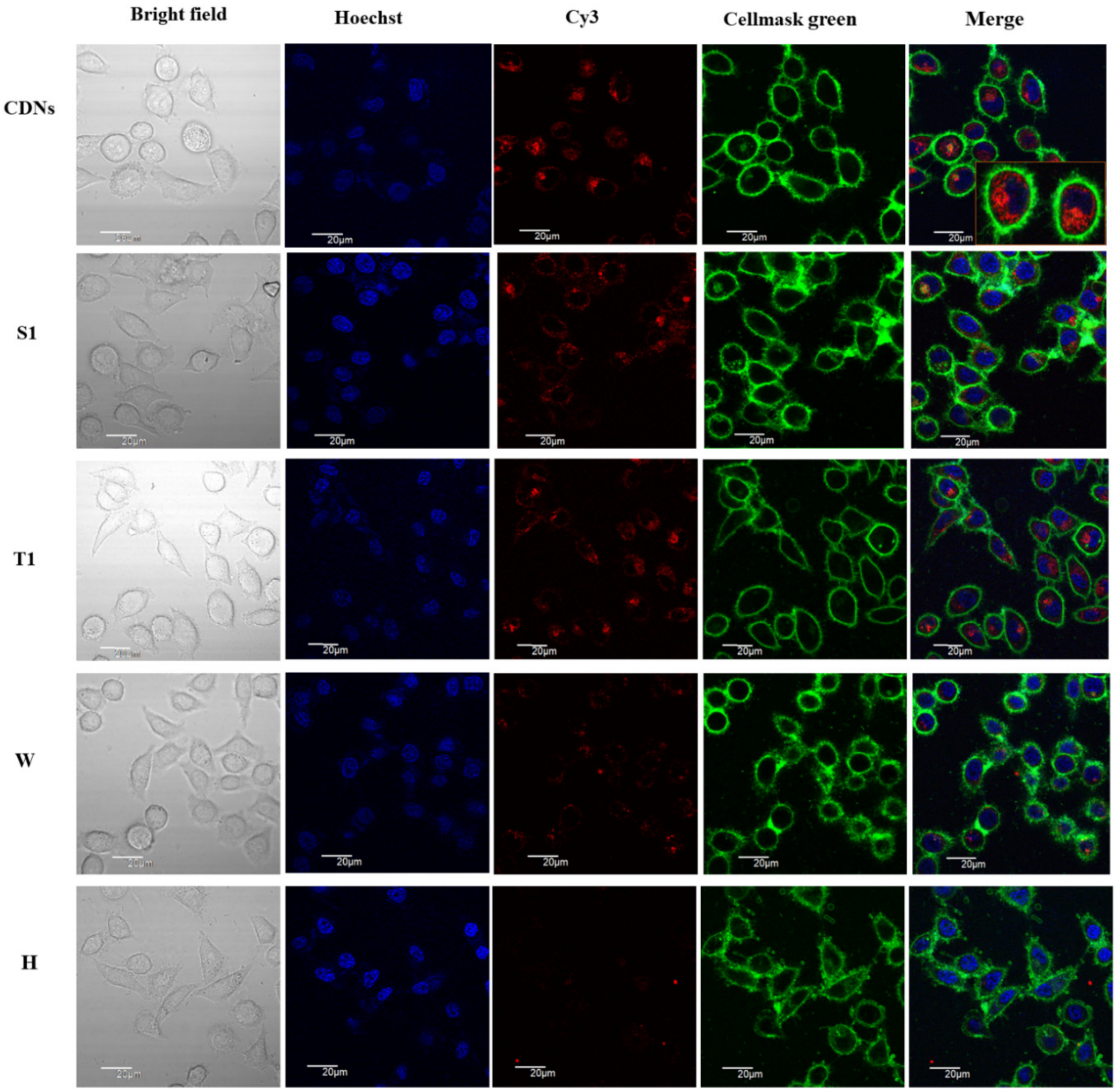

Figure S6. Cellular uptake analysed by confocal microscopy. Cy3 labelled samples of freshly prepared CDNs, lyophilized CDNs with sucrose (S1) and trehalose (T1), lyophilized CDNs without lyoprotectant (W) and heat-denatured CDNs (H) were incubated with HeLa cells for 6 hours. Nuclei and cell membrane of HeLa cells were stained with Hoechst 33342 dye and Cellmask green, respectively. Scale bars represent 20 µm.
